# Quantitative Imaging Parameters of Contrast-Enhanced Micro-Computed Tomography Correlate with Angiogenesis and Necrosis in a Subcutaneous C6 Glioma Model

**DOI:** 10.3390/cancers12113417

**Published:** 2020-11-18

**Authors:** Lízbeth Ayala-Domínguez, Enrique Pérez-Cárdenas, Alejandro Avilés-Salas, Luis Alberto Medina, Marcela Lizano, María-Ester Brandan

**Affiliations:** 1 Programa de Doctorado en Ciencias Biomédicas, Instituto de Investigaciones Biomédicas, Universidad Nacional Autónoma de México, Ciudad de México 04510, Mexico; ayalaliz@gmail.com; 2 Unidad de Investigación Biomédica en Cáncer INCan/UNAM, Instituto Nacional de Cancerología, Ciudad de México 14080, Mexico; medina@fisica.unam.mx; 3 Subdirección de Investigación Básica, Instituto Nacional de Cancerología, Ciudad de México 14080, Mexico; epc_incan@yahoo.com.mx; 4 Departamento de Patología, Instituto Nacional de Cancerología, Ciudad de México 14080, Mexico; aaviless@incan.edu.mx; 5 Departamento de Física Experimental, Instituto de Física, Universidad Nacional Autónoma de México, Ciudad de México 04510, Mexico; 6 Departamento de Medicina Genómica y Toxicología Ambiental, Instituto de Investigaciones Biomédicas, Universidad Nacional Autónoma de México, Ciudad de México 04510, Mexico

**Keywords:** angiogenesis, necrosis, C6 cells, quantitative imaging, micro-CT, contrast-enhanced imaging, relative blood volume, enhancement, iodine concentration

## Abstract

**Simple Summary:**

Contrast-enhanced (CE) X-ray imaging techniques have been used to assess angiogenesis in patients and animal models of cancer in order to overcome the limitations of histological quantification of angiogenesis, such as spatial and temporal heterogeneity of tumors. Some studies have compared the quantitative imaging parameters obtained with static and dynamic CE X-ray imaging techniques, but their association with histological biomarkers of angiogenesis has never been directly compared. This study aimed to provide such a comparison in a suitable animal model for the study of angiogenesis, namely, the subcutaneous C6 glioma model. We found an agreement among the quantitative imaging parameters obtained with these techniques, and we also found an association between a set of them with angiogenesis and necrosis descriptors. This set of quantitative imaging parameters demonstrated a high potential to describe angiogenesis and could be used to assess treatment response in further studies with this animal model.

**Abstract:**

The aim of this work was to systematically obtain quantitative imaging parameters with static and dynamic contrast-enhanced (CE) X-ray imaging techniques and to evaluate their correlation with histological biomarkers of angiogenesis in a subcutaneous C6 glioma model. Enhancement (E), iodine concentration (C_I_), and relative blood volume (rBV) were quantified from single- and dual-energy (SE and DE, respectively) micro-computed tomography (micro-CT) images, while rBV and volume transfer constant (K^trans^) were quantified from dynamic contrast-enhanced (DCE) planar images. C_I_ and rBV allowed a better discernment of tumor regions from muscle than E in SE and DE images, while no significant differences were found for rBV and K^trans^ in DCE images. An agreement was found in rBV for muscle quantified with the different imaging protocols, and in C_I_ and E quantified with SE and DE protocols. Significant strong correlations (Pearson *r* > 0.7, *p* < 0.05) were found between a set of imaging parameters in SE images and histological biomarkers: E and C_I_ in tumor periphery were associated with microvessel density (MVD) and necrosis, E and C_I_ in the complete tumor with MVD, and rBV in the tumor periphery with MVD. In conclusion, quantitative imaging parameters obtained in SE micro-CT images could be used to characterize angiogenesis and necrosis in the subcutaneous C6 glioma model.

## 1. Introduction

Angiogenesis is a mechanism of tumor vascularization characterized by the formation of new blood vessels from the pre-existing vasculature [1]. Microvessel density (MVD) is a surrogate biomarker of angiogenesis, it is quantified by histology and provides useful prognostic and predictive information for the management of cancer patients [2,3]. The main limitation of histological quantification of MVD is related to the temporal and spatial heterogeneity of tumors. In order to overcome this limitation, contrast-enhanced (CE) imaging techniques have been proposed to evaluate the angiogenic status of tumors in vivo, taking advantage of the immature and leaky nature of angiogenic vessels [4].

Several imaging modalities have been used to evaluate tumor vasculature in the clinical setting and to assess the potential of the imaging parameters to provide diagnostic, prognostic, and predictive information, such as computed tomography (CT) and magnetic resonance imaging (MRI), among others [4,5,6]. Dynamic contrast-enhanced MRI (DCE-MRI), dynamic susceptibility contrast-enhanced MRI (DSC-MRI), and perfusion CT are the most widely used techniques, and each one of them has advantages and disadvantages related to technical, biological, and clinical factors [4]. The relationship between the concentration of the contrast agent and the signal intensity in DCE-MRI and DCS-MRI is not linear and depends on the original tissue signal and on the parameters of the sequence used for image acquisition. On the contrary, signal intensity in contrast-enhanced (CE) X-ray imaging in general, including perfusion CT, is directly related to the attenuation properties of the tissues. Therefore, CE X-ray imaging techniques could provide a more direct interpretation of the imaging parameters in terms of their physiological meaning [4].

CE X-ray imaging techniques have been used to assess the association of quantitative imaging parameters with histological biomarkers of angiogenesis in preclinical studies with animals [7]. Dynamic contrast-enhanced (DCE) imaging with clinical CT scanners, i.e., perfusion CT, has been the most used CE X-ray imaging technique to study angiogenesis in animal models of cancer [8,9,10,11]. Static single- and dual-energy techniques (SE and DE, respectively) have been used with micro-CT or clinical CT scanners [12,13,14,15], or with planar imaging systems, such as in mammography [16]. Results from DCE studies are not consistent, since some have found a significant correlation between imaging and histological parameters of angiogenesis, while others have made evident that this association depends on how these parameters are quantified [8,9,13]. Another possible explanation of this variability in DCE studies could be related to the inadequate spatial resolution of clinical CT scanners for small animals imaging, which could affect the quantification of the arterial input function (AIF) required for kinetic analysis [17]. Static SE and DE techniques are less dependent on the spatial and temporal resolution of the scanners, but only a few preclinical studies are available with these protocols.

A few studies in cancer patients and one study in an animal model of glioma have addressed the comparison of quantitative imaging parameters obtained with SE, DE, and DCE techniques [6,18,19]; however, their potential to characterize tumor angiogenesis has never been directly compared. The aim of this work was to systematically quantify imaging parameters with standardized and optimized SE, DE, and DCE techniques, and to evaluate their correlation with histological biomarkers of angiogenesis in a subcutaneous C6 glioma model. This animal model is well suited for in vivo studies of angiogenesis, since its microvasculature has been previously described by imaging and histology [20,21,22,23]. Quantitative imaging parameters were obtained from SE and DE micro-CT images: enhancement (E), iodine concentration (C_I_), and relative blood volume (rBV). Volume transfer constant (K^trans^) and rBV were quantified using a DCE protocol based on planar projections, in a similar fashion as digital subtraction angiography. The correlation of these quantitative imaging parameters and percent necrosis (PN), proliferation index (PI), and MVD was evaluated.

## 2. Results

### 2.1. Qualitative Evaluation of CE X-ray Images of the Subcutaneous C6 Glioma Model

**Figure 1:**
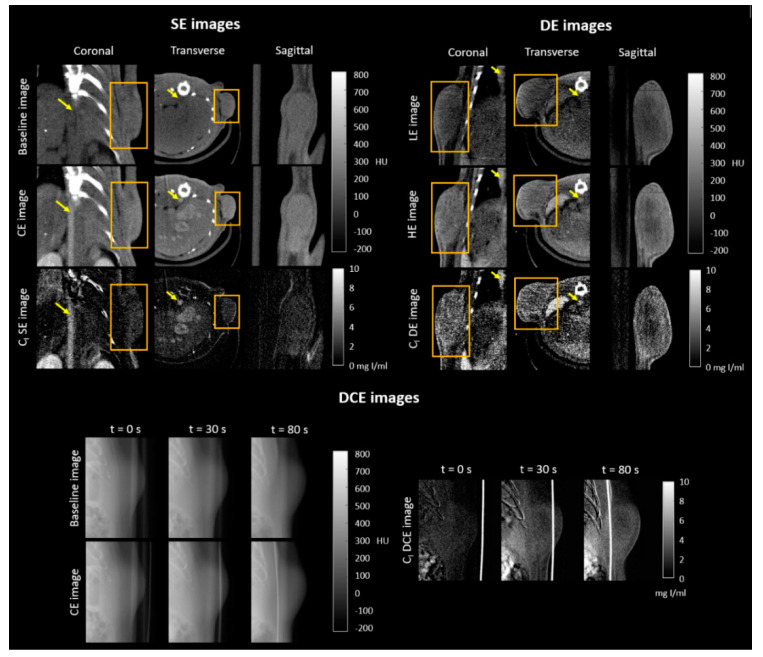
Qualitative evaluation of single-energy (SE) and dual-energy (DE) micro-computed tomography (micro-CT) images and dynamic contrast-enhanced (DCE) planar images of the subcutaneous C6 glioma model. Left upper panel: coronal, transverse, and sagittal views are shown for a baseline image, a contrast-enhanced (CE) image, and the iodine concentration (C_I_) SE subtracted image. Right upper panel: coronal, transverse, and sagittal views are shown for a low-energy (LE) image, a high-energy (HE) image, and the C_I_ DE subtracted image. Abdominal aorta and tumor are indicated by an arrow and square, respectively, in the coronal and transverse views. Only the tumors were depicted in the sagittal views. Bottom panel: Three acquisition times are shown for a baseline planar image, a CE planar image, and the C_I_ DCE subtracted image. The presence of the catheter used for the administration of the contrast agent was observed in the images.

Figure 1 shows different views of SE, DE, and DCE images of C6 glioma xenografts implanted in both flanks of Wistar rats. The left upper panel in Figure 1 shows baseline (pre-contrast), CE, and C_I_ SE micro-CT images; the right upper panel shows different views of low-energy (LE), high-energy (HE), and C_I_ DE micro-CT images; and the bottom panel shows baseline, CE, and C_I_ DCE planar images. CE SE and CE DCE images were obtained during continuous infusion of a clinical contrast agent; LE and HE images were obtained after the injection of a blood pool contrast agent. E SE and E DCE images were obtained after subtraction of the baseline image from the CE image, while E DE images were obtained after the weighted subtraction of the LE image from the HE image. E images are not shown since they are similar to C_I_ images under adequate windowing. C_I_ images (in mg of iodine per mL) were obtained after applying proper calibration functions to E images.

The abdominal aorta, liver vessels, kidneys, and tumor were visibly enhanced in the CE SE images in Figure 1 and were more clearly depicted in C_I_ SE images. Images of the bed and bone were eliminated from the C_I_ subtracted SE images, since image registration was performed before digital subtraction. In the C_I_ DE images, enhancement was observed in the tumor, abdominal aorta, spleen, and liver, as shown in Figure 1; bone removal was not observed in C_I_ DE images since the weighting factor for subtraction was chosen to eliminate tissue content, not bone [24]. A tumor section is shown in C_I_ DCE images in Figure 1; it was observed that tumor enhancement increased with time and that bone removal was more effective for the image acquired at *t* = 0 s.

### 2.2. Quantitative Evaluation of CE X-ray Images of the Subcutaneous C6 Glioma Model

**Figure 2:**
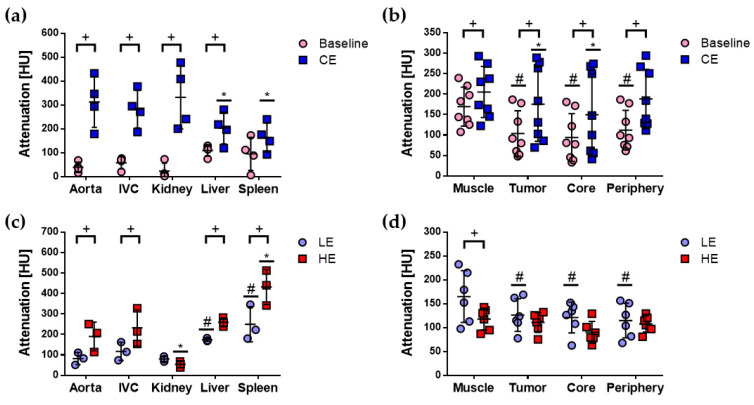
Quantitative evaluation of attenuation in SE and DE micro-CT images of the subcutaneous C6 glioma model. Mean and standard deviation are shown in scatter plots to compare attenuation values for baseline and CE images acquired with the SE protocol for (**a**) aorta, inferior vena cava (IVC), kidney, liver, and spleen and (**b**) muscle and tumor regions: complete tumor (tumor), central tumor (core), and peripheral tumor (periphery). Mean and standard deviation are shown in scatter plots to compare attenuation values for LE and DE images acquired with the DE protocol for (**c**) aorta, IVC, kidney, liver, and spleen and (**d**) muscle and tumor regions. Statistically significant differences (*p* < 0.05) were obtained with two-way ANOVA and Bonferroni’s multiple comparisons test and are indicated for each comparison: (+) baseline vs. CE or LE vs. HE; (#) aorta (or muscle) vs. volumes of interest (VOIs) in baseline and LE images; (*) aorta (or muscle) vs. VOIs in CE and HE images.

Attenuation, in Hounsfield units (HU), was evaluated in baseline and CE micro-CT images for the SE protocol, and in LE and HE micro-CT images for the DE protocol, for several volumes of interest (VOIs), namely, abdominal aorta, inferior vena cava (IVC), kidney cortex, liver, spleen, paraspinal muscle, and tumor regions (complete tumor (tumor), central tumor (core), and peripheral tumor (periphery)) as shown in Figure 2. Four animals (*n* = 8 tumors) were imaged with the SE protocol, and three animals (*n* = 6 tumors) with the DE protocol. Significant differences of attenuation values between baseline and CE images or between LE and DE images are indicated by (+) in Figure 2. Attenuation was higher in CE images for all VOIs, except spleen, while aorta, IVC, liver, spleen, and muscle showed significant differences between LE and DE images. Overall, these differences were related to the differential uptake of the contrast agents used with each imaging protocol.

The attenuation in the aorta was compared to the attenuation in IVC, kidney, liver, and spleen for baseline, CE, LE, and HE images. All mean values were similar in baseline images; significant differences were found in liver, spleen, and kidney in CE, LE, and HE images, and are indicated by (*) or (#) in Figure 2a,c. Explicitly, mean attenuation was significantly higher in the aorta than in liver and spleen in CE images, significantly lower in the aorta than in liver and spleen in LE images, and lower in the aorta than in spleen in HE images. Mean attenuation was also significantly higher in the aorta than in kidney in HE images. These differences were expected since it is known that the clinical contrast agent used with the SE protocol is cleared via the kidneys, while the blood pool contrast agent used with the DE protocol is cleared via the liver and spleen [7].

Significant differences were found between the attenuation in muscle and the tumor regions in baseline, CE, and LE images and are indicated by (*) or (#) in Figure 2b,d. These differences suggested that tumor regions can be distinguished from muscle from baseline, CE, and LE images; however, it must be noted that muscle showed similar or higher mean attenuation values than the tumor regions, which could prevent their visualization directly from these images.

**Figure 3:**
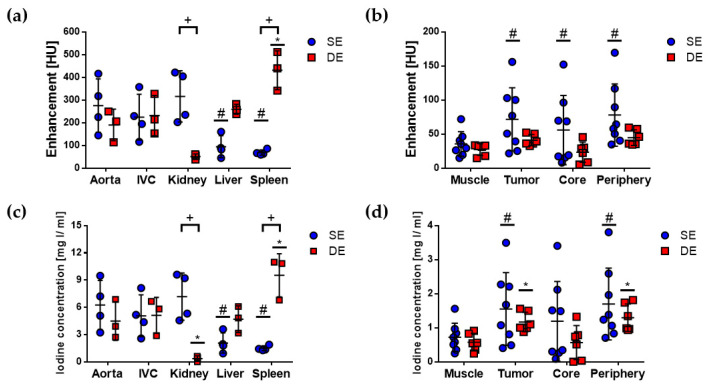
Quantitative evaluation of enhancement (E) and C_I_ in SE and DE micro-CT images of the subcutaneous C6 glioma model. Mean and standard deviation are shown in scatter plots to compare E in (**a**) aorta, IVC, kidney, liver, and spleen and (**b**) muscle and tumor regions: complete tumor (tumor), central tumor (core), and peripheral tumor (periphery), quantified in SE and DE subtracted images. Mean and standard deviation are shown in scatter dot plots to compare C_I_ in (**c**) aorta, IVC, kidney, liver, and spleen and (**d**) muscle and tumor regions, quantified in SE and DE subtracted images. Statistically significant differences (*p* < 0.05) were evaluated with two-way ANOVA and Bonferroni’s multiple comparisons test and are indicated for each comparison: (+) SE vs. DE, for all VOIs; (#) aorta (or muscle) vs. VOIs in SE images; (*) aorta (or muscle) vs. VOIs in DE images.

E and C_I_ values were quantified in the same VOIs as attenuation and are shown in Figure 3. Statistically significant differences of E and C_I_ mean values between SE and DE images were found for kidney and spleen, as indicated by (+), and were related to the differences in their elimination pathways. E and C_I_ mean values for the other VOIs were not significantly different among SE and DE images, which reflected the consistency of the different imaging protocols to quantify similar imaging parameters in the same animal model.

E and C_I_ mean values were significantly different between the aorta and liver and spleen in SE images, as indicated by (#), and between the aorta and spleen in DE images, as indicated by (*), in Figure 3a,c. C_I_ in the aorta was also significantly different from C_I_ in the kidney in DE images. It can be observed that there were marked differences in E and C_I_ values in the aorta among the different animals for both SE and DE protocols, which reflected the biological variability in this kind of studies. That is why it could be important to introduce a quantitative parameter such as rBV that takes this variability into account.

The comparison of E and C_I_ mean values between muscle and tumor regions yielded some differences between SE and DE images, particularly, significant differences between E in muscle and the tumor regions were only observed in SE images, as indicated by (#) in Figure 3b, while significant differences between C_I_ in muscle and the tumor regions were observed in both SE and DE images, as indicated by (#) and (*) in Figure 3d. This suggested that C_I_ could be a more sensitive parameter than E for distinguishing between muscle and the tumor regions with both imaging protocols. These results indicated that quantitative E and C_I_ images acquired with SE and DE protocols could allow better visualization of the tumor regions than attenuation images. This was inferred qualitatively from Figure 1, in which tumors and other enhanced structures were more clearly depicted in C_I_ images than in CE, LE, or HE images.

**Figure 4:**
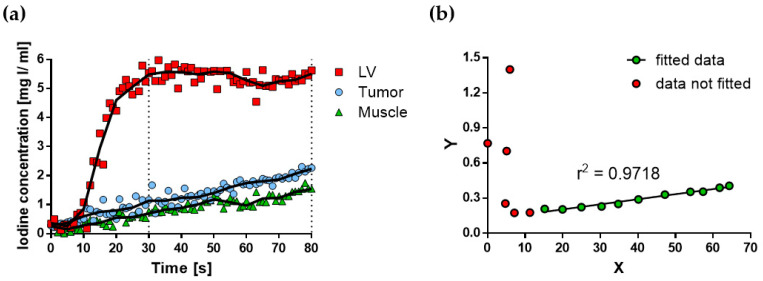
Quantitative evaluation of the DCE planar images of the subcutaneous C6 glioma model. (**a**) Time–C_I_ curves of the left ventricle (LV), tumor, and muscle, during the continuous infusion of a clinical contrast agent. Original data are shown (symbols), as well as smoothed data (solid lines). (**b**) Patlak analysis of the tumor data. Volume transfer constant (K^trans^) and relative blood volume (rBV) were quantified as the slope and the intercept, respectively, of the fitted line to data in the time interval of 30–80 s (green circles).

Two animals (*n* = 4 tumors) were imaged with the DCE protocol. DCE planar images (one per second) were analyzed in the time interval 0–80 s during the continuous infusion of the contrast agent and were used to obtain time–C_I_ curves of the left ventricle (LV), muscle, and tumor. Figure 4 shows representative time–C_I_ curves for one animal; a constant C_I_ was reached after the initial slope in the time–C_I_ curve of the LV, while a constant C_I_ increase was observed for tumor and muscle. Patlak analysis of the tumor data is shown in Figure 4b and it is described in Materials and Methods.

### 2.3. Quantification of rBV and K^trans^

**Figure 5:**
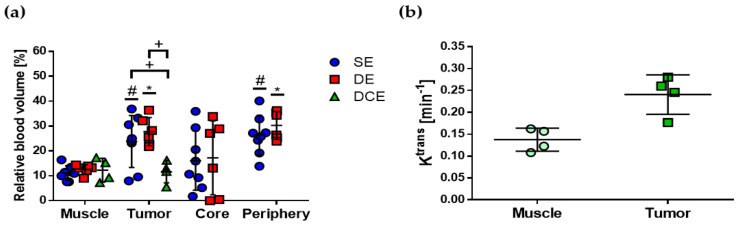
Quantitative imaging parameters from SE, DE, and DCE images of the subcutaneous C6 glioma model. (**a**) Mean and standard deviation are indicated in the scatter plot of rBV, which was quantified from SE (*n* = 8), DE (*n* = 6), and DCE (*n* = 4) images. (**b**) Mean and standard deviation are indicated in the scatter plot of K^trans^, which was quantified from DCE images (*n* = 4). Statistically significant differences (*p* < 0.05) were evaluated with two-way ANOVA and *t*-test for rBV and K^trans^, respectively, and are indicated for each comparison: (+) SE vs. DE, SE vs. DCE, DE vs. DCE; (#) muscle vs. VOIs in SE images; (*) muscle vs. VOIs in DE images.

The abdominal aorta was used as the AIF to quantify rBV from the C_I_ SE and C_I_ DE micro-CT images, while the LV was used as the AIF to quantify rBV and K^trans^ from kinetic analysis of time–C_I_ curves from DCE planar images. Figure 5 shows the results of the quantification of rBV and K^trans^ for these imaging protocols. The differences of rBV among the three imaging protocols were not statistically significant in muscle, central tumor, and peripheral tumor. In complete tumor, statistically significant differences were found in the rBV between DCE and the other protocols, as indicated by (+) in Figure 5a. These differences could be due to the inherent difficulties in evaluating the actual complete tumor in C_I_ DCE planar images.

A statistically significant difference was found between the mean rBV value in muscle and complete and peripheral tumor for SE and DE protocols, as indicated by (#) and (*) in Figure 5a, respectively, which was similar to the result for C_I_ shown in Figure 3d. For the central tumor, this difference with muscle was not significant, mainly due to the wide range in rBV and C_I_ values found in this tumor region. The difference in mean K^trans^ values between muscle and tumor was not statistically significant. Table 1 summarizes the results for E, C_I_, rBV, and K^trans^ evaluated in muscle and the tumor regions of the subcutaneous C6 glioma model and obtained with the CE X-ray imaging protocols explored in this work; significant differences with muscle are also indicated with (*). Together, these results suggested that C_I_ and rBV, quantified in SE and DE micro-CT images, allow better discernment between muscle and the tumor regions, both qualitatively and quantitatively, than attenuation, E, or the kinetic parameters quantified with DCE planar images.

### 2.4. Histological Validation of Quantitative Imaging Parameters

**Figure 6:**
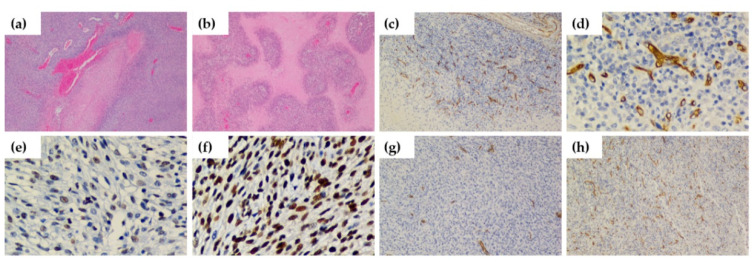
Histological characterization of the subcutaneous C6 glioma model. (**a**) Highly cellular tumors with pleomorphism and zones of coagulative necrosis lined by palisading neoplastic cells were observed in tissue sections of the tumors (hematoxylin and eosin (H&E), 100×). (**b**) Complex form of microvascular hyperplasia with peri-endothelial growth patterns (H&E, 100×). An increased number of microvessels were observed near necrotic areas in tissue sections of tumors, shown with magnifications of (**c**) 100× and (**d**) 400×. Representative immunohistochemical staining of proliferating cell nuclear antigen (PCNA) from tumors with (**e**) 50% proliferation index (PI) (400×) and (**f**) 95% PI (400×). Representative immunohistochemical staining of CD34 from tumors with (**g**) microvessel density (MVD) = 4.4 vessels/high-power field (HPF) (100×) and (**h**) MVD = 18.3 vessels/HPF (100×).

General histological features of the C6 glioma model were identified in tissue sections of tumors stained with hematoxylin and eosin (H&E) and are shown in Figure 6a,b. Figure 6a shows highly cellular tumors with pleomorphism and zones of coagulative necrosis lined by palisading neoplastic cells, and Figure 6b shows a complex form of microvascular hyperplasia with peri-endothelial growth patterns. Another characteristic feature of the C6 glioma model is shown in Figure 6c,d, in which tissue sections of tumors stained with an anti-CD34 antibody, used to identify microvessels and quantify MVD, show increased MVD at the periphery of necrotic areas.

Tumor characterization of the subcutaneous C6 glioma model was performed by evaluating PN, PI and MVD. Images e and f from Figure 6 show representative immunohistochemical staining of proliferating cell nuclear antigen (PCNA) from tumors with 50% and 95% PI, respectively. Images g and h from Figure 6 show representative immunohistochemical staining of CD34 from tumors with 4.4 vessels/high-power field (HPF) and 18.3 vessels/HPF, respectively. Table 2 summarizes the histological findings of the subcutaneous C6 glioma model for each imaging protocol; no statistically significant differences were found among the groups for each imaging protocol for these histological biomarkers. Additional images of tumors with the minimum and maximum values of PN, PI, and MVD for the SE, DE, and DCE imaging protocols are provided in Supplementary Materials Figures S1–S3, respectively.

**Figure 7:**
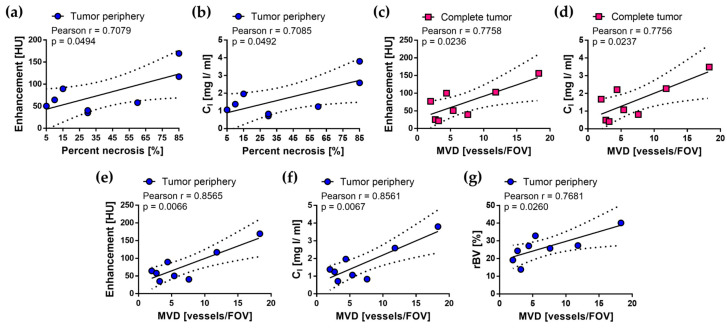
Histological validation of the quantitative imaging parameters from SE images of the subcutaneous C6 glioma model. Strong and significant correlations were found for seven sets of imaging and histological parameters: percent necrosis and (**a**) enhancement and (**b**) C_I_ at tumor periphery; MVD and (**c**) enhancement and (**d**) C_I_ in complete tumor; MVD and (**e**) enhancement, (**f**) C_I_, and (**g**) rBV at tumor periphery. Pearson *r* and *p*-value for each correlation are indicated in the graphs, as well as 95% confidence intervals (dotted lines).

Pearson correlation coefficient was calculated for pairs of histological biomarkers (PN, PI, and MVD) and quantitative imaging parameters (E, C_I_, rBV, and K^trans^) for the three tumor regions and for each imaging protocol to evaluate their association. Significant correlations (*p* < 0.05) were found for the pairs of parameters shown in Figure 7. No significant associations were found for imaging parameters quantified in DE or DCE images. For SE images, E and C_I_ in tumor periphery were associated with MVD and necrosis, E and C_I_ in the complete tumor with MVD, and rBV in the tumor periphery with MVD.

## 3. Discussion

The aim of this work was to systematically quantify radiological imaging parameters with SE, DE, and DCE techniques, and to evaluate their correlation with histological biomarkers of angiogenesis in a subcutaneous C6 glioma model. In order to obtain reliable quantitative imaging parameters, we used SE, DE, and DCE imaging protocols that were previously standardized and optimized [25,26,27]. Our results showed that CE X-ray imaging improved the visualization of the tumor regions. This was corroborated quantitatively for SE and DE images since C_I_ and rBV were significantly different between muscle and the tumor regions. A consistency of the quantitative imaging parameters was observed among the different techniques since similar values were found for E and C_I_ between SE and DE images in vascular structures, muscle, and tumor regions. Additionally, similar values were observed for rBV in muscle among SE, DE, and DCE images. Finally, we evaluated the association between the histological biomarkers of angiogenesis and the imaging parameters quantified in SE, DE, and DCE images and found a set of significant correlations for SE images.

The C6 glioma model resembles the histological features of human glioblastoma (GBM) [21,28,29], which presents necrotic areas surrounded by tumor cells, creating pseudopalisades [21]. Interestingly, it has been shown that these cells induce angiogenesis by the overexpression of vascular endothelial growth factor-A (VEGF-A) [20], which is the master regulator of angiogenesis [30]. Therefore, an increased MVD is generally found around necrotic areas. It has been observed that cells at the tumor periphery also overexpress VEGF-A, yielding a high MVD in this tumor region [28]. The histological features of the C6 glioma model were observed in tissue sections of the tumors evaluated in this work. Moreover, the differences between central and peripheral tumor were also observed in the CE and C_I_ images, and in the E, C_I_, and rBV values.

The use of subcutaneous versus orthotopic C6 glioma models has been widely debated and it has been demonstrated that both models have similar histological features, and microvessel morphology and permeability [31,32,33]. Among the advantages of the subcutaneous model versus the orthotopic model are the easier implantation of the cells, tumor volume assessment, and tumor resection. One disadvantage is the reduced time before tumor regression, which is governed by an alloimmune response against the implanted cells and could be an important limitation for survival studies [32]. Another disadvantage of the subcutaneous model is the lack of the blood–brain barrier (BBB) [33], which could yield different results among models in drug delivery studies, including CE imaging, depending on the chemical nature and molecular weight of the evaluated drugs.

Previous studies of the orthotopic C6 glioma model have used DE [14,15] or DCE imaging techniques [9,10,11], and general findings among them are in agreement with our observations in the subcutaneous C6 glioma model. Both animal models allowed to obtain CE X-ray images that improved lesion detection and reflected the heterogeneity in E, C_I_, or rBV values quantified in the different tumor regions, despite the presence of the BBB in the orthotopic model, which could be explained by the similarities in microvessel morphology and permeability between these two animal models [31].

Other studies have evaluated the quantitative imaging parameters with DCE [8], SE [13,16], and DE [12] imaging using different cell lines to generate subcutaneous tumors, and their general findings are also in agreement with our observations in the subcutaneous C6 glioma model. This observation is in accordance with Holash et al. [28], who demonstrated the consistency of vascular growth patterns between the orthotopic C6 glioma model and other animal models, as well as one human cancer. In another study, Ehling et al. [12] compared the quantitative imaging parameters of tumors obtained with several cell lines (four) with multiple angiogenic phenotypes, and only one imaging protocol. Interestingly, the rBV quantified for each of the four animal models was significantly associated with MVD (quantified with CD31), and this association was also significant when all the tumors were evaluated together [12]. These findings suggest that the quantitative imaging parameters obtained with CE X-ray imaging are capable of describing the angiogenic status of the animal model evaluated, regardless of the origin of the cell line or its angiogenic phenotype. In this manner, the subcutaneous C6 glioma model was expected to be equally useful as the orthotopic model or other cell lines to biologically validate the quantitative imaging parameters evaluated in this work. However, care must be taken when comparing different studies since the absolute value of the quantitative imaging parameters is highly dependent on the angiogenic phenotype of the evaluated cell line [12,28].

A few studies have compared the quantitative imaging parameters obtained with SE, DE, and DCE imaging protocols in order to elucidate their similarity [19] and their potential to provide diagnostic information [6] or to assess treatment response [18]. In this work, we compared the imaging parameters obtained with SE, DE, and DCE protocols and found a consistency among them, which is in agreement with previous findings in patients [6,19] and one animal model of cancer [18]. Kang et al. [19] evaluated patients with colorectal cancer and found significant correlations between C_I_ quantified in DE images and blood volume (BV) and permeability surface area product (PS) quantified in DCE images. Knobloch et al. [18] evaluated an animal model in which GS9L glioma cells were implanted in the foreleg of the animals and found significant correlations among imaging parameters in SE (attenuation), DE (C_I_), and DCE CT (blood flow (BF) and PS) images. The consistency among the imaging parameters found in our work suggested that if a change is observed in the quantitative parameters, it would be more likely related to a biological process than to a difference in its quantification, which is a major concern in quantitative imaging and it can be achieved by optimizing and standardizing the processes involved in image acquisition and analysis [34,35].

Despite the fact that the imaging parameters obtained from SE, DE, and DCE images have been compared in the studies mentioned above, to our knowledge, their association with histological biomarkers of angiogenesis has not been evaluated in the light of these comparisons. Moreover, only a few DE and DCE studies have evaluated the correlation of angiogenesis biomarkers and quantitative imaging parameters of the C6 glioma model. In this work, we evaluated the association of the imaging parameters with PN, PI, and MVD since they are relevant histological biomarkers in the clinical management of GBM [36,37].

We found significant positive correlations between PN and E and C_I_ at the tumor periphery in the SE images. These results are in apparent contradiction with Qi et al. [11], since they found a significant negative correlation between PN and BV and BF, quantified from DCE CT images of the C6 glioma model. We hypothesize that this discrepancy is related to the differences in the kinetic models used to quantify the imaging parameters. However, further studies are required in order to demonstrate this assumption. On the other hand, DCE-MRI has been widely used to differentiate necrosis induced by tumor progression and necrosis induced after radiation treatment in GBM animal models [38]. However, the association of PN with the MRI imaging parameters has not been widely investigated in GBM animal models. Zoula et al. [39] found a significant positive correlation between PN and the intensity of the lipid signal in proton magnetic resonance spectroscopy in an orthotopic C6 glioma model. In another study, Bradley et al. [40] also found a significant positive correlation between PN and K^trans^ (adjusted by fit failures) in the Hras5 animal model. Both results are in agreement with our findings for the SE imaging protocol, but they are not directly comparable due to the different nature of the signals used to form the images.

Liu et al. [14] found significant correlations between PI (obtained with anti-Ki67) and C_I_ quantified with a DE CT protocol, and suggested that areas with high PI increased the local blood flow and permeability. In studies with patients, a significant positive correlation has been found between several DCE-MRI imaging parameters and PI [41]. However, there is a lack of studies for the evaluation of tumor cell proliferation with MRI imaging in GBM animal models. Hou et al. [23] found a significant positive correlation between K^trans^ and PI (obtained with anti-PCNA) evaluated with DCE-MRI in an orthotopic C6 glioma model. In our study, we did not find significant correlations between PI (obtained with anti-PCNA) and any of the imaging parameters for the tumor regions evaluated with SE, DE, and DCE imaging protocols, probably because the viable tumor exhibited a limited range of PI values.

MVD has been associated with imaging parameters from DE and DCE images of the C6 glioma model [9,10,15]. Huang et al. [15] found a significant correlation between MVD (anti-CD105) and C_I_ quantified from DE CT images. Guan et al. [9] quantified BF and BV from DCE CT images and found significant correlations with MVD (anti-CD34). Lu et al. [10] also used a DCE CT technique and found a significant correlation between MVD (anti-CD105) and BF and BV. We found that MVD (anti-CD34) was associated with E and C_I_ quantified in the complete tumor, and also with E, C_I_, and rBV quantified in the tumor periphery of SE micro-CT images. However, we found no significant associations of the DE and DCE imaging parameters and MVD, which could be related to the limitations found in our study. Several DCE-MRI studies have evaluated the association of the quantitative kinetic parameters and MVD in the orthotopic C6 glioma model. Hou et al. [23] found a significant positive correlation between K^trans^ and MVD (anti-CD34), which is in agreement with our findings with the SE imaging protocol. Recently, DCE-MRI has also been used to evaluate the different growth patterns described in GBM [22].

The first limitation in our study was the small sample size for each imaging protocol, which limited the statistical power of the study and thus it might be difficult to extrapolate our results. Second, there were technical limitations in the implementation of DE and DCE imaging protocols. For the DE protocol, only a reduced kilovoltage range was available in our scanner (X-ray tube voltage 30–45 kV), which was not optimal for selecting radiation qualities with effective energies around the K-edge of iodine, and thus the contrast obtained in DE images was limited. Another limitation in the DE imaging protocol was the reduced iodine concentration of the blood pool contrast agent that we used (50 mg I/mL) compared with the iodine concentration of the clinical contrast agent used in SE and DCE protocols (300 mg I/mL), which could explain the similar attenuation and enhancement values observed between muscle and the tumor regions in DE images.

The major limitation to implement DCE protocols in our scanner was the temporal resolution, since a complete micro-CT image requires at least a 2-min acquisition. Therefore, we used planar projections in order to achieve a high temporal resolution that allowed us to image the fast kinetics of the distribution of the clinical contrast agent within the animals; however, the superimposition of the tissues in the planar images made difficult the quantification of C_I_ in the entire tumor volume. The most common administration technique of the contrast agent in clinical perfusion studies is bolus injection. However, we used continuous infusion instead of bolus injection to improve the visualization of the enhanced tissues in the DCE planar images, since a constant increment of C_I_ in the tissues can be achieved with continuous infusion compared with a higher, but rapidly decaying, C_I_ achieved with the bolus injection [42]. The kinetic parameters quantified from the Patlak analysis of the delayed phase of time–C_I_ curves, either after bolus injection or during continuous infusion of the contrast agent, are expected to be similar, since the C_I_ quantified in the tissues is independent of the administration technique in the delayed times of image acquisition [42].

Another limitation in our study was related to the radiation dose inherent to X-ray imaging. The absorbed dose could cause a damage to the animals if it is not properly quantified and optimized; moreover, it can also bias the study by affecting the development of the tumor under evaluation [7]. In order to limit the effects of the dose delivered in our studies, we previously optimized the radiological parameters for image acquisition that provided the highest image quality and the lowest radiation dose to the animals [25]. The radiation dose to water measured at the isocenter for the CE X-ray imaging protocols used in our study ranged from 213 mGy to 680 mGy and it was below the lethal dose LD_50/30_ for mice (5.0–7.6 Gy) [43].

Despite these limitations, the present study provided useful insight on the study of angiogenesis with CE X-ray imaging, particularly in the subcutaneous C6 glioma model. Future studies could explore the potential of C_I_ and rBV in SE images to provide prognostic and predictive information by assessing treatment response, as well as to provide diagnostic information such as to distinguish between benign and malignant tumors in suitable animal models of cancer.

## 4. Materials and Methods

### 4.1. Subcutaneous C6 Glioma Animal Model

C6 glioma cells (ATCC, Manassas, VA, USA) were cultured in RMPI 1640 medium (GIBCO, Thermo Fisher Scientific, Waltham, MA, USA), supplemented with 10% fetal bovine serum, at 37 °C with 5% CO_2_. Tumors were induced in immunocompetent male Wistar rats (average body weight ± standard deviation = 263 ± 21 g) by subcutaneous inoculation of 3 × 10^6^ C6 cells suspended in 200 µL of phosphate buffered saline (PBS), into their left and right flanks. Animals were kept in a pathogen-free environment and fed with autoclaved food and water ad libitum. Prior to imaging, each animal was anesthetized with isoflurane (3% in 100% oxygen), and then the right external jugular vein was catheterized for contrast agent administration with a heparinized PE10 polyethylene tube (Scientific Commodities Inc., Lake Havasu City, AZ, USA) and a syringe pump (KDS100, KD Scientific Inc., Holliston, MA, USA). CE X-ray images of the animals were acquired 14 ± 3 days post-inoculation of the C6 cells (mean ± standard deviation). All experimental procedures with the animals were reviewed and approved by the Ethics Committee and the Institutional Committee for Animal Welfare of the National Institute of Cancerology, Mexico, where all the experiments were carried out (approval number: (018/051/IBI) (CEI/1294/18)). All efforts were made to minimize animal suffering and to reduce the number of animals used in the experiments.

### 4.2. Albira ARS Micro-CT Scanner

The micro-CT scanner of the trimodal PET/SPECT/CT Albira ARS preclinical system (Bruker Corporation, Billerica, MA, USA) was used for image acquisition. Tomographic images were reconstructed into a 750 × 750 × 657 matrix (100 µm voxel size) with the simultaneous iterative reconstruction technique (SIRT 2D) implemented in-house with the ASTRA toolbox in Matlab R2018b (The MathWorks Inc., Natick, MA, USA), running on a Dell Precision Workstation M4800 with Intel Core i7-4810MQ CPU and NVIDIA Quadro K1100M GPU. A calibration to HU was performed on each reconstructed micro-CT image using the attenuation value for water for each imaging protocol.

### 4.3. Imaging Protocols

SE, DE, and DCE acquisition protocols were previously optimized and standardized for small animal imaging with the Albira ARS micro-CT scanner [25,26,27]. The radiological parameters for image acquisition (X-ray tube voltage (kV), current (mA), additional filter, and number of projections) were chosen as the ones which provided the highest contrast-to-noise ratio and the lowest radiation dose; and the reconstruction algorithm was chosen in terms of quantitative image quality metrics. Optimal iodine dose, injection volume, and time for image acquisition after the injection of the contrast agents were also standardized.

#### 4.3.1. SE Protocol

SE micro-CT images were acquired with 45 kV, 0.8 mA, and 400 projections. A baseline (pre-contrast) image of the animal was acquired; then, a second CE image was acquired during continuous infusion of a clinical contrast agent (Omnipaque 300, GE Healthcare, Wauwatosa, WI, USA; average dose = 2.4 mg of iodine/g of body weight (b.w.), infusion rate = 0.5 mL/min). Total radiation dose to water measured at the isocenter was 680 mGy for this protocol.

#### 4.3.2. DE Protocol

LE micro-CT images were acquired with 30 kV, 0.8 mA, and 250 projections, while HE micro-CT images were acquired with 45 kV, additional molybdenum filter (25 µm thick, 99.9% purity), 0.8 mA, and 250 projections. HE image was acquired approximately 12 min after the administration of a blood pool contrast agent (Fenestra VC, MediLumine Inc., Montreal, QC, Canada; average dose = 0.4 mg of iodine/g b.w.); then, LE image was acquired immediately after the molybdenum filter was removed. For this protocol, dose to water at the isocenter was 213 mGy.

#### 4.3.3. DCE Protocol

The first 180 projections of a micro-CT image with 45 kV, 0.8 mA, and 1000 projections over 360° (one projection per second) were acquired as part of the DCE protocol. This method was chosen because it provided better temporal resolution than acquiring planar images directly (all at 0°) or a complete micro-CT image, which required large acquisition times due to the technical configuration of the scanner. Baseline projections were acquired first and then, CE projections were acquired during the continuous infusion of a clinical contrast agent (Omnipaque 300, GE Healthcare, Wauwatosa, WI, USA; average dose = 1.2 mg of iodine/g b.w., infusion rate = 0.5 mL/min). Total radiation dose to water at the isocenter for the DCE protocol was 340 mGy.

### 4.4. Image Analysis

#### 4.4.1. Image Subtraction

SE and DCE images were obtained after subtraction of the baseline image from the CE image, while DE images were obtained after weighted subtraction of HE and LE images: DE = HE − αLE, where α = 0.55 was the weighting factor chosen to eliminate most of the unenhanced tissue content in the DE image [24]. Baseline images were registered to CE images (and LE images to HE images) with affine transformation before subtraction [44]. Proper calibration functions were obtained with iodinated phantoms [45] and applied to subtracted SE, DE, and DCE images in order to obtain C_I_ images.

#### 4.4.2. Quantification of Imaging Parameters in SE and DE Micro-CT Images

Attenuation, in HU, was evaluated in baseline and CE micro-CT images for the SE protocol, and in LE and HE micro-CT images for the DE protocol. Amide software [46] was used to draw ellipsoidal VOIs in abdominal aorta, inferior vena cava, kidney, liver, spleen, paraspinal muscle, and tumor regions, i.e., complete tumor (tumor), central tumor (core), and peripheral tumor (periphery). Table S1 indicates the average size of the VOIs used to quantify the imaging parameters in SE and DE micro-CT images. The central tumor was defined as an ellipsoid with half the dimensions of the complete tumor. The imaging parameters in the peripheral tumor were averaged from four VOIs that were drawn in the tumor volume outside the central tumor. All VOIs were placed in the same position for each animal, and the same size of the VOI was used for a given organ or tissue among all animals. E and C_I_ values were quantified in E and C_I_ images, respectively; and the same VOIs used to quantify attenuation were used to quantify E and C_I_ for a given animal. The C_I_ quantified in the abdominal aorta was used to obtain the rBV values: rBV = 100 × (C_I_/C_I,aorta_).

#### 4.4.3. Quantification of Imaging Parameters in DCE Planar Images

Patlak analysis was used to quantify rBV and K^trans^ from time–C_I_ curves. This method considers a two-compartmental model in which the contrast agent transits from the intravascular space to the extravascular-interstitial space, as dictated by K^trans^, and then accumulates in the extravascular-interstitial space, described by rBV, considering that no back flux exists, i.e., the second compartment is irreversible [47]. This theoretical assumption can only be achieved under two experimental conditions: a time after bolus injection of the contrast agent, or during its continuous infusion. The latter condition was used in this work.

Time–C_I_ curves were obtained for three VOIs (left ventricle, triceps muscle, and complete tumor) from the C_I_ DCE images with ImageJ software [48]. To quantify the kinetic parameters, the following expression was obtained from the time–C_I_ curves (1) [47]:

(1) $$\frac{C_{I,\mathrm{tissue}}\left(t\right)}{C_{I,\mathrm{AIF}}\left(t\right)}=K^{\mathrm{trans}}\frac{\int_{T_{1}}^{T_{2}}C_{I,\mathrm{AIF}}\left(t\right)\mathrm{dt}}{C_{I,\mathrm{AIF}}\left(t\right)}+\mathrm{rBV},$$

in which C_I,tissue_(t) and C_I,AIF_(t) represent the time–C_I_ curves of tissue (muscle or tumor) and AIF (LV), respectively. It can be noted that Equation (1) has the form of a linear equation, in which the kinetic parameters K^trans^ and rBV represent the slope and intercept, respectively. Therefore, an estimation of the kinetic parameters can be obtained after a linear regression analysis of Equation (1) in the time interval T_1_–T_2_, in which the condition of the irreversible compartment is met. Only data from the time interval 30–80 s were considered for linear regression analysis of Equation (1), as illustrated in Figure 4.

### 4.5. Histological Analysis

Tumors were excised, processed, and embedded in paraffin. Consecutive tumor sections of 4 µm thickness were cut and mounted on positively charged slides. Immunohistochemistry was performed to evaluate PN; PI was evaluated with an anti-PCNA primary antibody (sc-56, dilution 1:500; Santa Cruz Biotechnology, Inc., Dallas, TX, USA); and MVD was evaluated with an anti-CD34 primary antibody (ab81289, dilution 1:500; Abcam, Cambridge, UK). Positive staining was detected with the DAB HRP Brown detection system (Bio SB, Santa Barbara, CA, USA). Negative controls corresponded to incubation without primary antibodies; human tonsil and vascular tumor were used as positive controls for anti-PCNA and anti-CD34, respectively. PN was evaluated as the percentage of necrotic tissue contained in the tumor, PI was estimated as the percentage of positive cells to anti-PCNA in the viable tumor, and MVD was quantified as the mean value of the vessel count in 10 high-power fields of hot spots [49]. A Nikon ECLIPSE E200 optical microscope (Nikon Instruments Inc., Melville, NY, USA) with a 10× eyepiece and 10× and 40× objective lens was used.

### 4.6. Statistical Analysis

Data in the scatter plots were expressed as mean ± standard deviation, and data in the tables were expressed as mean ± standard error of the mean. GraphPad Prism 6 (GraphPad Software, Inc., San Diego, CA, USA) was used to perform all statistical analyzes. Attenuation, E, C_I_, and rBV were compared among protocols with two-way analysis of variance (ANOVA) and Bonferroni’s multiple comparisons test. K^trans^ values were compared with a two-sided *t*-test. Histological parameters among the groups for each imaging protocol were compared with one-way ANOVA and Tukey’s multiple comparisons test. Pearson correlation coefficient was estimated to evaluate the association of the histological biomarkers to the quantitative imaging parameters. A *p*-value less than 0.05 was considered as statistically significant.

## 5. Conclusions

In this study, we systematically quantified radiological imaging parameters with SE, DE, and DCE protocols in the subcutaneous C6 glioma model. We corroborated that CE X-ray imaging improved lesion detection, both qualitatively and quantitatively. The quantitative imaging parameters evaluated in this study demonstrated consistency among the different imaging protocols, despite their wide inter- and intra-tumor variability. Moreover, significant associations were found among the imaging parameters quantified in SE images and the histological biomarkers of angiogenesis: E and C_I_ in tumor periphery were associated with MVD and necrosis, E and C_I_ in the complete tumor with MVD, and rBV in the tumor periphery with MVD. In this manner, this work provided evidence of the potential use of the imaging parameters quantified in SE micro-CT images as descriptors of angiogenesis and necrosis in the C6 glioma model.

## Figures and Tables

**Table 1:** Quantitative imaging parameters in muscle and the tumor regions of the subcutaneous C6 glioma model obtained with single-energy (SE), dual-energy (DE), and dynamic contrast-enhanced (DCE) images.

Imaging Protocol	Imaging Parameter	Muscle	Tumor	Core	Periphery
SE (*n* = 8)	E (HU)	35.5 ± 6.3	71.6 ± 16.3 *	55.8 ± 17.9 *	77.9 ± 16.1 *
	C_I_ (mg I/mL)	0.72 ± 0.14	1.55 ± 0.37 *	1.19 ± 0.41	1.69 ± 0.37 *
	rBV (%)	11.1 ± 1.0	23.8 ± 3.6 *	16.1 ± 4.2	26.3 ± 2.8 *
DE (*n* = 6)	E (HU)	27.2 ± 3.4	41.1 ± 3.3	23.4 ± 6.0	44.7 ± 4.6
	C_I_ (mg I/mL)	0.57 ± 0.10	1.17 ± 0.11 *	0.56 ± 0.20	1.29 ± 0.16 *
	rBV (%)	12.6 ± 0.7	27.7 ± 2.3 *	17.2 ± 6.0	30.2 ± 2.2 *
DCE (*n* = 4)	rBV (%)	12.2 ± 2.3	11.6 ± 2.2		
	K^trans^ (min^−1^)	0.13 ± 0.01	0.24 ± 0.02		

E, enhancement; C_I_, iodine concentration; rBV, relative blood volume; K^trans^, volume transfer constant. Values are expressed as mean ± standard error of the mean; * *p* < 0.05, compared to muscle.

**Table 2:** Histological characterization of subcutaneous C6 glioma tumors that were imaged with single-energy (SE), dual-energy (DE), and dynamic contrast-enhanced (DCE) protocols.

Imaging Protocol	Percent Necrosis (%)	Proliferation Index (%)	MVD (Vessels/HPF)
SE (*n* = 8)	38.1 ± 10.5	68.7 ± 8.4	6.9 ± 1.9
DE (*n* = 6)	28.3 ± 7.6	70.8 ± 8.2	3.5 ± 1.0
DCE (*n* = 4)	26.2 ± 9.4	78.7 ± 6.5	3.9 ± 1.5

MVD, microvessel density; HPF, high-power field. Values are expressed as mean ± standard error of the mean.

## Supplementary Materials

The following are available online at https://www.mdpi.com/2072-6694/12/11/3417/s1, Figure S1: Representative histological samples of the subcutaneous C6 glioma model evaluated with the SE imaging protocol, Figure S2: Representative histological samples of the subcutaneous C6 glioma model evaluated with the DE imaging protocol, Figure S3: Representative histological samples of the subcutaneous C6 glioma model evaluated with the DCE imaging protocol, Table S1: Average VOI size and estimated number of pixels used for the quantification of imaging parameters for several tissues/organs in SE and DE micro-CT images.
