# Supplementary material for: Quantitative Imaging Parameters of Contrast-Enhanced Micro-Computed Tomography Correlate with Angiogenesis and Necrosis in a Subcutaneous C6 Glioma Model
Source: Cancers (Basel). 2020 Nov 18;12(11):3417. doi: 10.3390/cancers12113417 (PMC7698719; doi:10.3390/cancers12113417)
Supplement: Supplementary file 1 [file cancers-12-03417-s001.pdf]

Supplementary Materials: Quantitative Imaging Parameters of Contrast-Enhanced Micro-Computed Tomography Correlate with Angiogenesis and Necrosis in a Subcutaneous C6 Glioma Model

Lízbeth Ayala-Domínguez, Enrique Pérez-Cárdenas, Alejandro Avilés-Salas, Luis Alberto Medina, Marcela Lizano and María-Ester Brandan

Figure S1. Representative histological samples of the subcutaneous C6 glioma model evaluated with the SE imaging protocol.

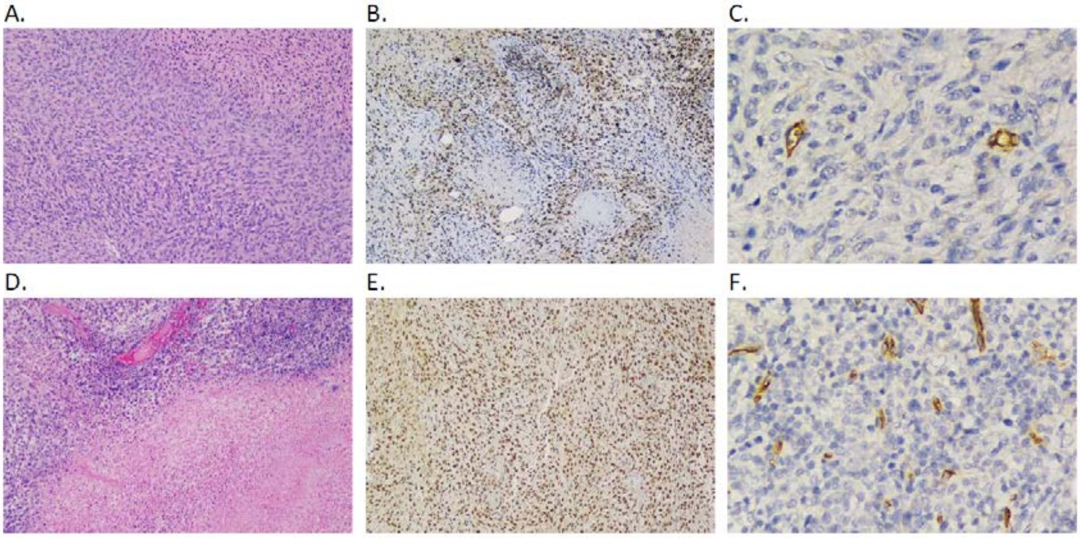

For this group, the lowest percent necrosis (PN) was (**A**) 5% and the highest was (**B**) 85%; the lowest proliferation index (PI) was (**C**) 40%, and the highest was (**D**) 95%; the lowest microvessel density (MVD) was (**E**) 2.0 vessels/high-power field (HPF), and the highest was (**F**) 18.3 vessels/HPF. MVD images are shown at 400× magnification, all other images are shown at 100× magnification.

Figure S2. Representative histological samples of the subcutaneous C6 glioma model evaluated with the DE imaging protocol.

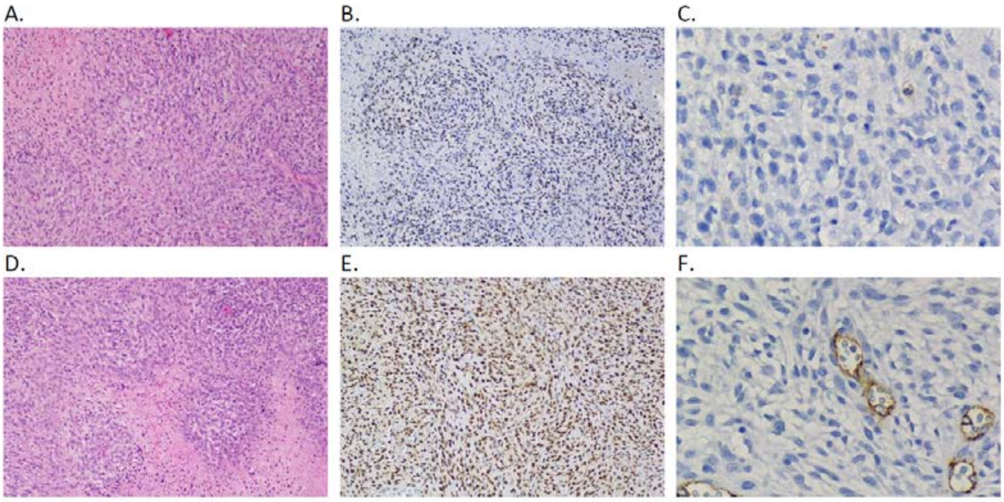

For this group, the lowest PN was (**A**) 15% and the highest was (**B**) 60%; the lowest PI was (**C**) 50%, and the highest was (**D**) 95%; the lowest MVD was (**E**) 0.9 vessels/field of view (FOV), and the highest was (**F**) 7.6 vessels/HPF. MVD images are shown at 400× magnification, all other images are shown at 100× magnification.

Figure S3. Representative histological samples of the subcutaneous C6 glioma model evaluated with the DCE imaging protocol.

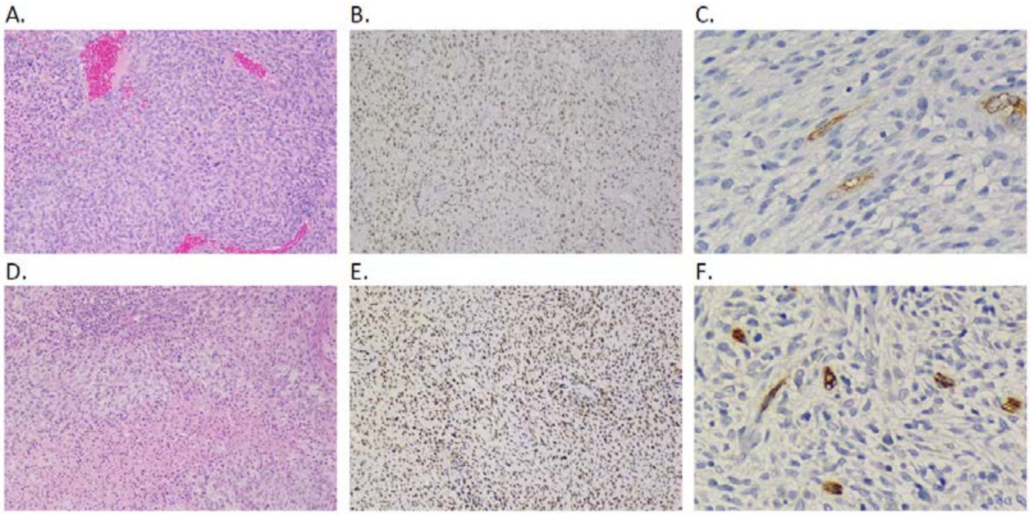

For this group, the lowest PN was (**A**) 5% and the highest was (**B**) 50%; the lowest PI was (**C**) 60%, and the highest was (**D**) 90%; the lowest MVD was (**E**) 1.3 vessels/field of view (FOV), and the highest was (**F**) 6.3 vessels/HPF. MVD images are shown at 400× magnification, all other images are shown at 100× magnification.

Table S1. Average VOI size and estimated number of pixels used for the quantification of imaging parameters for several tissue/organs in SE and DE micro-CT images.

	SE Imaging Protocol		DE Imaging Protocol	
**Tissue/Organ**	**Volume (mm³)**	**Estimated Number of Voxels**	**Volume (mm³)**	**Estimated Number of Voxels**
Abdominal aorta	0.5 ± 0.0	713.3 ± 6.1	0.5 ± 0.0	719.0 ± 4.2
IVC	1.8 ± 0.0	2191.0 ± 2.8	1.8 ± 0.0	2196.5 ± 26.2
Kidney	0.5 ± 0.0	717.5 ± 3.5	0.5 ± 0.0	721.0 ± 2.8
Liver	1.8 ± 0.0	2188.0 ± 2.8	1.8 ± 0.0	2185 ± 4.9
Spleen	0.5 ± 0.0	716.3 ± 5.5	0.5 ± 0.0	721.0 ± 4.2
Muscle	0.5 ± 0.0	718.8 ± 6.1	0.5 ± 0.0	723.5 ± 4.9
Complete tumor	334.2 ± 126.8	349,180.5 ± 130,381.6	694.3 ± 94.8	718,005.3 ± 96,071.6
Central tumor	41.8 ± 15.8	45,570.3 ± 16,752.2	86.8 ± 11.9	92,762.5 ± 12,123.8
Peripheral tumor	2.0 ± 1.1	2400.3 ± 1308.5	1.8 ± 1.6	2135.9 ± 1834.0

Values are expressed as mean ± standard deviation.

© 2020 by the authors. Licensee MDPI, Basel, Switzerland. This article is an open access article distributed under the terms and conditions of the Creative Commons Attribution (CC BY) license (http://creativecommons.org/licenses/by/4.0/).
